# Biobanking for Viral Hepatitis Research

**DOI:** 10.3389/fmed.2019.00183

**Published:** 2019-08-20

**Authors:** Erwin Ho, Stijn Van Hees, Sofie Goethals, Elke Smits, Manon Huizing, Sven Francque, Benedicte De Winter, Peter Michielsen, Thomas Vanwolleghem

**Affiliations:** ^1^Department of Gastroenterology and Hepatology, Antwerp University Hospital, Antwerp, Belgium; ^2^Laboratory of Paediatrics and Experimental Medicine, University of Antwerp, Antwerp, Belgium; ^3^Biobank Antwerp, Antwerp University Hospital, Antwerp, Belgium; ^4^Department of Gastroenterology and Hepatology, Erasmus Medical Center, Rotterdam, Netherlands

**Keywords:** viral hepatitis, SLiMs, biobank, screening, immunology, B cells

## Abstract

**Introduction:** Viral hepatitis is a worldwide, important health issue. The optimal management of viral hepatitis infections faces numerous challenges. In this paper, we describe how biobanking of biological samples derived from viral hepatitis patients collected both in-hospital and during community outreach screenings provides a unique collection of samples.

**Materials and Methods:** All samples and materials were provided with a study code within the SLIMS system Study protocols and an informed consent form were approved by the Antwerp University Hospital/University of Antwerp Ethical Committee. Systematic biobanking was initiated in October 2014. Collected sample types include: (1) serum and plasma of all newly diagnosed HBV, HCV, HDV, and HEV positive patients; (2) left-over serum and plasma samples from all PCR analyses for HBV and HCV performed in the context of routine clinical care; (3) left-over liver tissue not needed for routine histological diagnosis after liver biopsy; and (4) additional virus-specific, appropriate sample types using a scientific rationale-based approach. A community outreach screening program was performed in three major Belgian cities. Serum, EDTA, Tempus Blood RNA and BD Vacutainer CPT were collected. CPT tubes were centrifuged on-site and mononuclear cells collected within 24 h.

**Results:** Concerning community screening: 298 individuals supplied all 4 sample types. Samples were stored at −150°C and were logged in the biobank SLIMS database. Samples were used for HBV-related immunological and biomarker studies. DNA isolated from plasma samples derived from chronic HBV patients was used to investigate Single Nucleotide Polymorphism rs 1790008. Serum samples collected from chronic hepatitis C patients were used to assess the efficacy of HCV treatment. Peripheral Blood Mononuclear Cells (PBMC) isolated from chronic HBV patients and healthy controls were used for different immunological study purposes. Virus isolated from biobanked stool of a chronic hepatitis E patient was used to establish a mouse model for Hepatitis E infections, allowing further HEV virology studies.

**Conclusion:** The establishment of a biobank with samples collected both in-hospital and during community-outreach screening resulted in a unique, continuously expanding collection of biological samples which provides an excellent platform for prompt answers to clinically and translational relevant research questions.

## Introduction

Viral hepatitis is a worldwide, important health issue, mostly caused by five different primary hepatotropic viruses: The Hepatitis A Virus (HAV), Hepatitis B Virus (HBV), Hepatitis C virus (HCV), Hepatitis Delta Virus (HDV), and Hepatitis E Virus (HEV). Infected patients are at increased risk of developing liver-related events, including liver failure, liver cirrhosis and hepatocellular carcinoma, ultimately culminating in liver-related death (1–3). As such, viral hepatitis accounts for an estimated 1.45 million deaths annually, 90% of which are attributed to chronic HBV and HCV infections (3). Importantly, this number is on the rise, ranking viral hepatitis among the most important causes of death worldwide (3).

HBV and HCV replicate non-cytopathically in human hepatocytes. As such, liver damage caused by both viruses incurs primarily through host immune responses (4–6). Chronic infection develops in 10% of adult HBV and approximately 80% of HCV infected patients (4–6). The immunopathogenesis of both infections is, however, not fully understood (1, 4–6). As of now, HCV is curable, but HBV is not. The Hepatitis B Virus forms a stable genomic “reservoir” within the nucleus of infected hepatocytes. It integrates part of its genetic code in the host genome and forms covalently closed circular DNA (cccDNA), which acts as a mini-chromosome. Current standard of care treatment suppresses viral replication, but fails to clear the genomic “reservoir” (1). Additionally, HDV infections are seen in up to 5% of chronic HBV patients, leading to more aggressive liver disease, not seldom presenting with liver complications before the fourth decade of life (7).

HEV infections are mostly self-limiting in immunocompetent hosts, but chronic infections may develop in immunosuppressed or HIV coinfected hosts. Little is known on its pathogenesis (8). Management of chronic infections involves lowering the dosage of immunosuppressants with addition of ribavirin treatment if needed, which results in viral clearance in up to 80% of the infected patients (8).

Clearly, the optimal management of viral hepatitis infections faces numerous challenges. In this paper, we describe how biobanking of biological samples derived from viral hepatitis patients and healthy controls collected both in-hospital and during community outreach screenings provides a unique collection of samples that can be used to investigate unanswered questions on the pathogenesis of viral hepatitis, and to optimize management thereof. We report the quality metrics, organization, output variables, the unique logistics, planning and execution associated with biobanking for viral hepatitis research. Subsequently, we show an overview of how biobanking at the Antwerp University Hospital has resulted in novel insights relating to viral hepatitis infections over the last 5 years.

## Methods

### General Considerations

Funding from the CMI program (Center for Medical Innovation) from the Flemish Government and existing biobanking infrastructure for oncology (Tumorbank@UZA part of the Belgian Virtual Tumorbank funded by the National Cancer Plan) allowed for the establishment of storage of biological samples for hepatotropic diseases, including samples collected for the study of viral hepatitis. As such, the established biobank is a disease-specific, hospital-integrated and community-based biobank.

The biological samples are managed by trained biobank personnel to ensure samples are handed, registered and stored according to an established biobank quality management system (QMS).

Important aspects of this QMS concerning sample maintenance include:

- Processing of samples by dedicated biobank personnel via Standard Operating Procedures (SOPs)
- Proper identification and traceability of samples via 2D barcode labeling of samples encoded in a sample management database (SLIMS, Genohm SA, Lausanne, Switzerland)
- Registration of important pre-analytical date/time stamps such as collection, reception, centrifugation, fractionation and storage in SLIMS
- Use of SPREC coding (9)
- Inclusion and exclusion criteria for uptake of samples in the biobank via fixed decision trees
- Regular checks of the defined critical dataset in SLIMS

The protocol was carried out in accordance with the recommendations of Good Clinical Practice, approved by the Antwerp University Hospital/University of Antwerp Ethical Committee (EC 15/21/227), with written informed consent from all subjects. All subjects gave written informed consent in accordance with the Declaration of Helsinki. The informed consent allows sampling and storage for blood, urine, fecal and liver materials from hepatology outpatient and inpatients clinics.

The established biobank comprises three different categories of samples: (1) prospectively collected samples during in-hospital based biobanking; (2) samples collected during outreach community screenings; (3) left-over serum samples from routine PCR analyses in the clinical laboratory and left-over liver tissue samples not needed for routine histological diagnosis at the department of pathology. Informed consent was obtained from all patients from whom samples of the first two categories were collected. For category 1, informed consent was obtained during outpatient or inpatient hospital care by attending medical staff. Presumed consent was applicable to samples of category 3. Presumed consent is based on Belgian law where it is stated that the use of leftover human materials is allowed for diagnostic and research purposes (19-12-2008, “Law pertaining the acquisition and use of human materials for medical use in humans or in scientific research”). These statues and reference to the applicable law are written in the patient admission information flyer.

Samples of category 1 and 2 are reserved for primary use within a predefined timeframe by the investigators mentioned in the ethical committee approval for biobanking. Upon termination of the timeframe for primary use, these samples can be accessed by all researchers, including external researchers, upon approval of both the Ethical Committee and Biobank council. The concept of primary use does not apply to samples of Category three. These samples can be accessed immediately by all researchers upon ethical committee and biobank council approval.

During outreach community screenings, individuals were provided with Simplified Chinese and Traditional Chinese informed consent forms and information brochures, and additional translation support was provided on-site. These forms were approved by the Ethical Committee as part of the biobanking protocols. Prior to these community sessions, Q&A sessions were held to communicate the objectives of the study, the purpose of biobanking and the conditions of confidentiality/traceability (coding) of results and samples.

### Setting Up an In-Hospital Biobank for Non-tumor Samples

**Figure 1:**
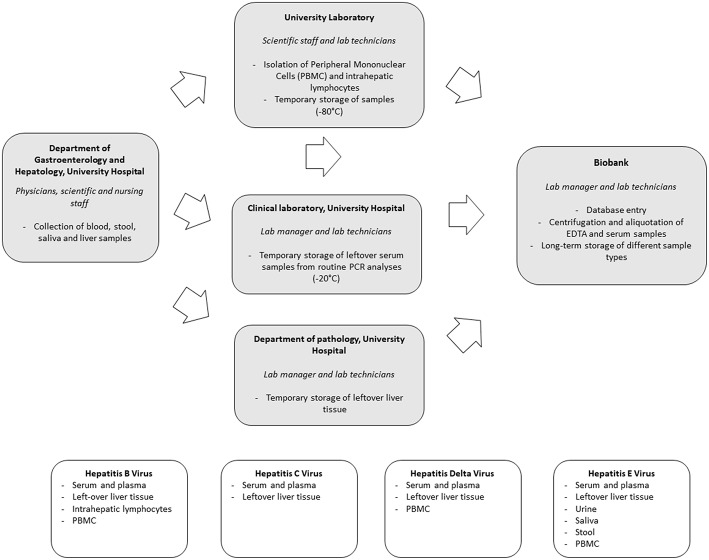
Sample flow and types of samples collected during hospital-based biobanking.

Preparations for in-hospital biobanking consisted of the integration of an extra option in the blood analysis request forms, the creation of a workflow for the acquisition of informed consents as well as regular exchanges between all parties involved to discuss the optimal sample flow. Systematic biobanking was subsequently initiated in October 2014. Collected sample types include: (1) serum and plasma of all newly diagnosed HBV, HCV, HDV, and HEV positive patients; (2) left-over serum and plasma from all PCR analyses for HBV and HCV performed in the context of routine clinical care; (3) left-over liver tissue not needed for routine histological diagnosis after liver biopsy and (4) additional virus-specific, appropriate biological sample types using a scientific rationale-based approach. An overview of the sample flow and collected sample types per virus is depicted in Figure 1.

Biobanking of serum and plasma samples of newly diagnosed patients is requested by the physician through the electronic blood analysis request forms. Blood is then sampled by the nursing staff and sent to the central biobank for centrifugation, aliquotation and storage through an in-house pneumatic tube system. Collaboration with the clinical laboratory allowed for the collection of left-over serum and plasma samples of all HBV and HCV PCR analyses performed for routine clinical care purposes. Samples are temporarily stored at −20°C at the clinical laboratory and then transferred in batch to the central biobank.

Hepatitis viruses all infect human hepatocytes. Unraveling what happens at the site of infection, namely the liver, is of utmost importance to understand the complex interplay between virus and host. Left-over tissue not needed for routine clinical histological evaluation provides a highly valuable resource of samples in this regard. We therefore set up a routine flow to collect and store left-over material in a standardized way.

A thorough understanding of the immune responses against hepatitis viruses requires a close collaboration between clinicians, laboratory personnel and biobank. We optimized a workflow for both intrahepatic as peripheral lymphocyte flowcytometric analyses. In select cases a part of the left-over liver tissue not needed for routine histological diagnosis is put in cell culture medium. The latter is then transferred to the University Laboratory for isolation of intrahepatic lymphocytes using a Fluorescence Activation Cell Sorting (FACS) based approach. Isolated intrahepatic lymphocytes are stored at −150°C in the central biobank. In addition, peripheral blood mononuclear cells (PBMC) are collected. Sampling of heparinized blood is requested by scientific staff through the electronic blood analysis forms. Blood is taken by nursing staff and transferred to the University Laboratory for isolation of PBMC by Ficoll-Hypaque density centrifugation. Isolated samples are temporarily stored at −80°C at the Antwerp University laboratory before transfer to −150°C freezers at the central biobank. Temperature monitoring is part of a hospital-wide system.

When compared to HBV and HCV, a unique aspect of HEV infection is the fecal-oral transmission route, especially for genotype 1 infections. The virus is readily detectable in human stool and monitoring of viremia in stool has proven to be an important tool in the management of chronic HEV infections (8). To further characterize different aspects of Hepatitis E infections, collection and biobanking of a wide range of body fluids, including saliva, stool and urine in addition to blood samples, was initiated. Left-over samples of stool not needed for routine clinical monitoring of HEV viremia, are stored at −80°C. In addition, also PBMC, urine and saliva are collected through nursing staff.

### Biobanking During Outreach Screening Projects

Asians have a higher seroprevalence of HBV infection, and presumably of HCV infection as well (10–12). This population is known to be difficult to reach, and epidemiological data in the Belgian-Asian/Chinese migrant population is lacking. While many screening studies have been performed in diaspora settings, biobanking has not. Biobanking during outreach community screening would constitute a unique “control” group: apart from disease-specific information in Hepatitis B surface Antigen (HBsAg) positive Asians, HBsAg negative persons in the same target population would provide an excellent control group with similar socio-demographic characteristics. These individuals have a high chance to be exposed to HBV but would not have been afflicted with HBV. This control group is typically lacking in previous biobanking efforts, where they are recruited from hospital or research environments, but not from the same community with high HBV prevalence.

**Figure 2:**
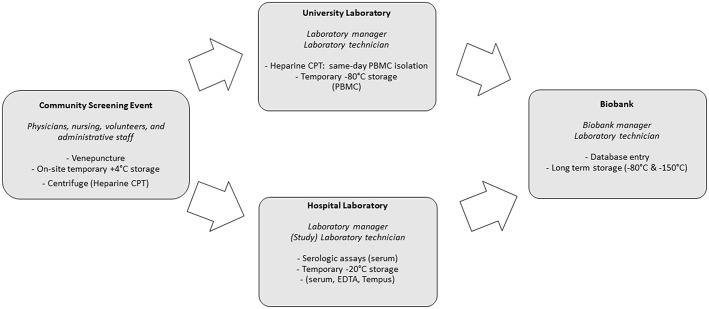
Sample flow and roles in community screening and biobanking.

Preparations for the community screenings and biobanking were executed simultaneously. This required a coordinated effort from screening staff (administrative, paramedic and medical), research laboratory staff, the hospital laboratory and the biobank itself. Additionally, community leaders and volunteers were crucial in providing preparatory, logistical and linguistic support during screening and biobanking. Figure 2 provides an overview of the activities performed by these different entities. Central SLIMS labeling, as previously mentioned, was provided. To facilitate the logistics of biobanking, standardized sachets with all required materials for screening and biobanking (including informed consent and request forms) were provided and labeled using the code registered in SLIMS.

Screenings were organized in three major Belgian cities: Antwerp, Brussels and Leuven between 9 a.m. and 5 p.m.

Serum, EDTA, Tempus Blood RNA (Applied Biosystems Tempus Blood RNA tube, 3 mL) and, for screenings organized in Antwerp, BD Vacutainer CPT (Mononuclear Cell Preparation Tubes, 4 mL) were collected during screening events. CPT tubes were centrifuged on-site for 15 min at 1,500 relative centrifugal force. CPT tubes were transported two or three times each session (depending on amount of samples and time) to allow for the University laboratory to perform the procedure to extract peripheral blood mononuclear cells (PBMCs) in a timely manner and prevent loss of cells by cell death. Serum, EDTA and Tempus were temporarily stored on-site at +4°C (cooling elements) and after the end of each session, in the clinical laboratory, at −20°C. Within 2 days, serum, EDTA, Tempus and PBMCs were subsequently stored in the biobank at −150°C. Additionally, plasma left-overs from CPT tubes were also stored.

Venepuncture testing for HBsAg, anti-HBc and anti-HCV was performed at the Antwerp University Hospital laboratory (Elecsys HBsAg II, anti-HBc, anti-HCV, Roche Diagnostics GmbH, Mannheim, Germany). Additional funding for community screening and biobanking was obtained from grants (Roche Diagnostics, Gilead Life Sciences Inc., Bristol-Myers Squibb, Sandoz).

## Results

### Number of Collected Samples

The number of samples stored in and retrieved from the biobank are shown in Tables 1, 2: samples obtained using informed consent or using presumed consent (hospital based) and community screening are shown. Biobanking from hospital sources amounted to a total of 10,419 samples (2,116 patients), community sourced samples to a total of 4,136 (462 persons). Retrievals were used for study purposes, in accordance to subsequent, Ethics Committee approved protocols. Forty-seven non-conformities were logged from 2015 to 2018 (2, 7, 21 and 17 in 2015, 2016, 2017, and 2018, respectively). These storage failures were due to erroneous sample withdrawal, pre-storage Turn-Around Time (TAT) violation, insufficient data collection/wrong identification, insufficient sample volume or the incorrect use of sample recipients.

**Table 1a:** Hospital-based sampling (informed consent).

	**2015**		**2016**		**2017**		**2018**	
**Sample type**	**Stored**	**Retrieved**	**Stored**	**Retrieved**	**Stored**	**Retrieved**	**Stored**	**Retrieved**
Serum	110	0	875	23	1,552	8	1,280	1
EDTA plasma	53	0	561	1	900	0	542	0
EDTA buffy coat non-viable	18	0	216	0	370	0	221	0
EDTA red blood cell	16	0	220	0	365	0	0	0
PBMC	0	0	62	0	139	13	80	12
Stool	0	0	2	1	5	0	7	0
Saliva	0	0	0	0	0	0	40	0
Urine	0	0	0	0	0	0	2	0
Intrahepatic lymphocytes	0	0	0	0	0	0	42	0
Total	197	0	1,936	25	3,331	21	2,214	13
Patients: 616								

*EDTA, Ethylenediaminetetraacetic acid tube; PBMC, peripheral blood mononuclear cells*.

**Table 1b:** Hospital-based sampling (presumed consent/leftover samples).

	**pre-2014**		**2014**		**2015**		**2016**		**2017**		**2018**	
**Sample type**	**Stored**	**Retrieved**	**Stored**	**Retrieved**	**Stored**	**Retrieved**	**Stored**	**Retrieved**	**Stored**	**Retrieved**	**Stored**	**Retrieved**
Serum	151	0	287	0	785	0	105	6	183	0	480	0
Liver tissue	576	0	41	0	37	0	35	0	32	0	29	0
Total	727	0	328	0	822	0	140	6	215	0	509	0
Patients: 1,500													

**Table 2:** Community-based sampling.

	**Stored**	**Retrieved**					
**Sample type**	**Total**	**Total**	**At sampling**	**2015**	**2016**	**2017**	**2018**
Serum, prime	421	421	0	0	0	0	0
Serum, aliquots	1,654	46	0	0	8	38	0
EDTA	159	0	0	0	0	0	0
Tempus	458	8	0	0	8	0	0
PBMC	217	19	0	0	8	11	0
CPT (plasma leftover)	299	238	0	238	0	0	0
Saliva	467	426	426	0	0	0	0
Dried blood spots	461	420	420	0	0	0	0
Total	4,136	1,578	846	238	24	49	0
Patients: 462							

*EDTA, Ethylenediaminetetraacetic acid tube; PBMC, peripheral blood mononuclear cells; Tempus, RNA blood collection tube; CPT, Cell Preparation Tube (for PBMC sampling)*.

## Cost Analysis

CMI structural funding for hepatotropic disease biobanking amounted to € 98,560. Personnel, operational, storage, database, QC and administrative costs were covered using these funds. Community sampling involved additional costs—these are shown in Table 3.

**Table 3:** Community-based biobanking: costs (in euros).

Personnel	Nursing staff	3, 634.0
	Administrative assistant	1, 157.7
	Language services	160.0
	Study coordinator	1, 719.8
	Physicians	4, 366.8
	Total	11, 038.3
Logistics	Blood tubes, venepuncture materials	4, 737.2
	Event logistics (location rent, catering, etc.)	500.0
	Communication costs	1, 211.0
	Total	6, 448.2
Overall cost		17, 486.5

## Discussion

Using a unique combination of outreach screening-based and hospital-based biobanking we were able to establish a continuously expanding collection of biological samples that enables a prompt answer to several relevant clinical and translational research questions in the field of viral hepatitis.

Numerous challenges arose during the execution of the project. Both types of biobanking required a different approach with inherently also different challenges. As for community outreach screening-based biobanking, despite the uniformity in data entry, labeling and storage, high personnel input from all participating entities is necessary to ensure success (Table 4). Additionally, a single coordinator is needed to ensure continuity and to remedy and track logistical and quality variance, for instance; traffic delayed CPT Heparine shipments from the screening locations to the university laboratory. This staff member had initially been planned to also perform screening, but personnel redundancy was quickly activated to ensure CPT Heparine transport could continue, whilst also being able to continue screening activities. By design, sample complexity was kept at a minimum, but the latter issue illustrates that PBMC collection in particular proved to be challenging during community outreach screening-based biobanking.

**Table 4:** Staff and tasks involved in the preparation and execution of biobanking during on-site screenings.

	**Task**	**Preparatory/support**	**On-site/during screening**
Administrative staff	Registration, on-site logistics	1	1
Paramedical staff	Venepuncture	1	2-3
Study coordinator		1	1
Medical staff	Informed consent, information	2	2
Volunteers	Translation, community coordination	4	5-10
Hospital laboratory	Serological testing, temporary storage	3	2
University laboratory	PBMC isolation and temporary storage	2	3-4
Biobank	Database, labeling, storage, sample processing and QA	3	1

Community screenings typically scale from tens of samples to hundreds or even thousands (13). In our experience, ±100 is an upper limit that our clinical and research laboratories could handle for sample processing and testing within turnaround times (TAT) that fall within performance characteristics. Serum samples, e.g., which needed to be tested for HBV and HCV serology, arrived in bulk. The platform which was used (Roche Diagnostics Elecsys, Modular) was not designed to rapidly run such a large number of samples. Additionally, the samples arrived after the screening event had ended, plus, some events were held on weekends. Thus, staffing at the clinical laboratory was lower than during weekdays. Despite these challenges, analytical TATs were within 5 days.

Hospital based biobanking presented different challenges (14, 15). Staffing and logistics are less time and resource intensive, as systems to obtain, process and store samples are already in place on-site. However, samples from healthy controls (including those who are not necessarily negative for a specific disease) are not collected during hospital based biobanking as opposed to screening/community settings. Patient recruitment in a hospital setting is more specific, and dedicated study coordinators need to monitor, often complex, inclusion criteria.

**Figure 3:**
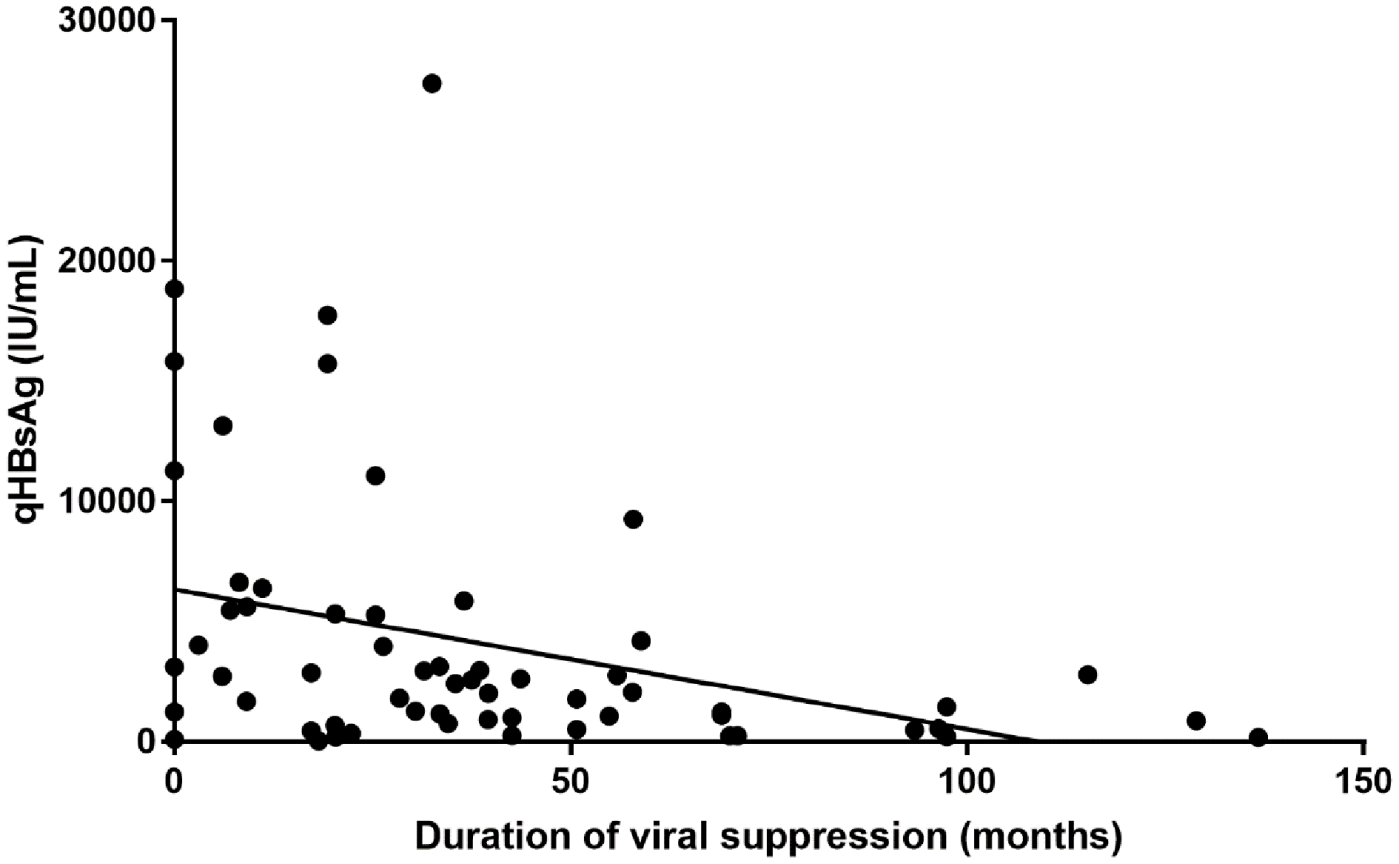
HBsAg quantification compared to duration of treatment induced viral suppression (in months).

Biobanked serum and plasma has so far been used for different purposes; one of which was the quantification of Hepatitis B surface Antigen levels in chronic hepatitis B patients. HBsAg quantification (“qHBsAg”) provides extra information in terms of the natural history of a person chronically infected with HBV. In our center, we have observed that qHBsAg slowly declines when patients are treated with nucleoside/nucleotide analog antiviral therapy (Figure 3). Recent literature suggests that qHBsAg levels may guide physicians in their decision to interrupt long-term antiviral treatment for chronic hepatitis B (10).

DNA isolated from plasma samples derived from chronic HBV patients was used in an international multicentre study to investigate the prevalence of a Toll-Like Receptor 7-specific Single Nucleotide Polymorphism (SNP) rs 1790008 in a large group of chronic HBV infected patients (*n* = 1,054) and healthy individuals (*n* = 231). The SNP was almost exclusively detected in Caucasian subjects and was much more prevalent in healthy Caucasian females when compared to HBV infected Caucasian females, suggesting that the SNP might provide protection against chronic HBV infection in this population (11). Serum samples collected from chronic hepatitis C patients were used in an international clinical trial to assess the efficacy of an 8-week treatment regimen of ledipasvir/sofosbuvir in HCV genotype 4 infected patients. Among a total of 39 included patients, 6 (of whom 2 were patients at the Antwerp University Hospital) did not meet the primary study endpoint, being HCV RNA negative at 12 weeks after therapy. Retrospective phylogenetic analyses on biobanked samples revealed that 4/6 of these patients had been reinfected (12).

**Figure 4:**
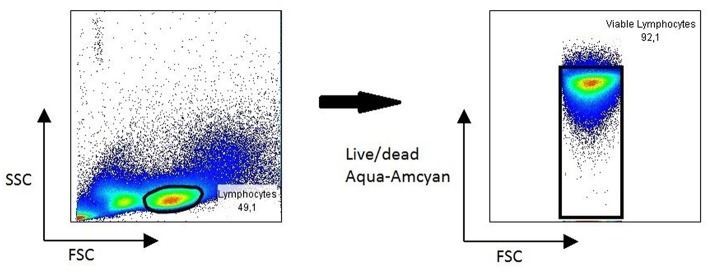
Representative FACS plot showing viability of lymphocytes in PBMC 3 years after collection.

PBMC isolated from chronic HBV patients and healthy controls showed excellent viability 3 years after isolation (Figure 4), allowing for use for different immunological study purposes. In one study, paired serum and PBMC samples of chronic Hepatitis B patients were used to study Hepatitis B specific B cell responses. Results revealed a strong association of a potent Hepatitis B-core specific B cell response with clinical parameters in both treated and untreated patients (16). In another study, the global B cell transcriptome was profiled in chronic HBV infected patients and compared to healthy controls using a systems biology approach. Peripheral B cells of chronic HBV patients showed clinical phase dependent transcriptome alterations and proved to be very different from intrahepatic B cells on a transcriptome level (17).

Virus isolated from biobanked stool of a chronic hepatitis E patient was successfully used to establish a mouse model for Hepatitis E infections, allowing further HEV virology studies (18, 19). Interestingly, using biobanked urine samples, we discovered that HEV RNA can be detected in urine samples.

Of final note, the higher amount of retrieved samples from community biobanking is largely due to requirements in study protocols. Going forward, biobank procedures have been put in place to facilitate third-party use of samples. A biobank committee (with principal investigators of studies that collected samples in the biobank) will process and evaluate requests on scientific merit, logistical feasibility and ethical considerations.

## Conclusion

In conclusion, in this chapter we described how the establishment of a biobank with samples collected both in-hospital and during community-outreach screening, resulted in a unique, continuously expanding collection of biological samples which provides an excellent platform for prompt answers to clinically and translationally relevant research questions. This information may guide other centers in setting up similar projects in possibly different contexts.

## Data Availability

The datasets generated for this study are available on request to the corresponding author.

## Ethics Statement

The protocol was carried out in accordance with the recommendations of Good Clinical Practice, approved by the Antwerp University Hospital/University of Antwerp Ethical Committee (EC 15/21/227), with written informed consent from all subjects. All subjects gave written informed consent in accordance with the Declaration of Helsinki. The informed consent allows sampling and storage for blood, urine, fecal and liver materials from hepatology outpatient and inpatients clinics.

## Author Contributions

EH and SV contributed equally to the work as shared-first authors. SG provided data and revisions of the text. PM and TV provided drafting and revisions of the text. All other authors listed (ES, MH, SF, and BD) have made also made substantial, direct and intellectual contribution to the work, and approved it for publication.

### Conflict of Interest Statement

The authors declare that the research was conducted in the absence of any commercial or financial relationships that could be construed as a potential conflict of interest.

## References

[1] Yuen MF Chen DS Dusheiko GM Janssen HLA Lau DTY Locarnini SA. Hepatitis B virus infection. Nat Rev Dis Prim. (2018) 4:18035. 10.1038/nrdp.2018.35 29877316

[2] Van Hees S Michielsen P Vanwolleghem T. Circulating predictive and diagnostic biomarkers for hepatitis B virus-associated hepatocellular carcinoma. World J Gastroenterol. (2016) 22:8271–82. 10.3748/wjg.v22.i37.8271 27729734 PMC5055858

[3] Stanaway JD Flaxman AD Naghavi M Fitzmaurice C Vos T Abubakar I. The global burden of viral hepatitis from 1990 to 2013: findings from the Global Burden of Disease Study 2013. Lancet. (2016) 388:1081–8. 10.1016/S0140-6736(16)30579-7 27394647 PMC5100695

[4] Chigbu DI Loonawat R Sehgal M Patel D Jain P Hepatitis C virus infection: host(-)virus interaction and mechanisms of viral persistence. Cells. (2019) 8:E376 10.3390/cells8040376 31027278 PMC6523734

[5] Bertoletti A Ferrari C. Adaptive immunity in HBV infection. J Hepatol. (2016) 64 (1 Suppl):S71–s83. 10.1016/j.jhep.2016.01.026 27084039

[6] Maini MK Gehring AJ. The role of innate immunity in the immunopathology and treatment of HBV infection. J Hepatol. (2016) 64 (1 Suppl):S60–70. 10.1016/j.jhep.2016.01.028 27084038

[7] Wranke A Pinheiro Borzacov LM Parana R Lobato C Hamid S Ceausu E. Clinical and virological heterogeneity of hepatitis delta in different regions world-wide: The Hepatitis Delta International Network (HDIN). Liver Int. (2018) 38:842–50. 10.1111/liv.13604 28963781

[8] Kamar N Izopet J Pavio N Aggarwal R Labrique A Wedemeyer H Hepatitis E virus infection. Nat Rev Dis Prim. (2017) 3:17086 10.1038/nrdp.2017.86 29154369

[9] Betsou F Bilbao R Case J Chuaqui R Clements JA De Souza Y. Standard PREanalytical Code Version 3.0. Vancouver, BC: Biopreservation and biobanking (2018). 10.1089/bio.2017.0109 PMC11708182 29377712

[10] Liu J Li T Zhang L Xu A. The role of Hepatitis B surface antigen in nucleos(t)ide analogues cessation among asian chronic hepatitis B patients: a systematic review. Hepatology. (2018). 10.1002/hep.30474 30561829

[11] Buschow SI Biesta PJ Groothuismink ZMA Erler NS Vanwolleghem T Ho E. TLR7 polymorphism, sex and chronic HBV infection influence plasmacytoid DC maturation by TLR7 ligands. Antiv Res. (2018) 157:27–37. 10.1016/j.antiviral.2018.06.015 29964062

[12] Boerekamps A Vanwolleghem T van der Valk M van den Berk GE van Kasteren M Posthouwer D. 8 weeks of sofosbuvir/ledipasvir is effective in DAA-naive non-cirrhotic HCV genotype 4 infected patients (HEPNED-001 study). J Hepatol. (2019) 70:554–7. 10.1016/j.jhep.2018.10.032 30527953

[13] Chen Z Chen J Collins R Guo Y Peto R Wu F. China Kadoorie Biobank of 0.5 million people: survey methods, baseline characteristics and long-term follow-up. Int J Epidemiol. (2011) 40:1652–66. 10.1093/ije/dyr120 22158673 PMC3235021

[14] Olson JE Ryu E Johnson KJ Koenig BA Maschke KJ Morrisette JA. The mayo clinic biobank: a building block for individualized medicine. Mayo Clin Proc. (2013) 88:952–62. 10.1016/j.mayocp.2013.06.006 24001487 PMC4258707

[15] Mooser V Currat C. The lausanne institutional biobank: a new resource to catalyse research in personalised medicine and pharmaceutical sciences. Swiss Med Weekly. (2014) 144:w14033. 10.4414/smw.2014.14033 25474562

[16] Vanwolleghem T Grooshuismink A Kreefft K Hung M Novikov N Boonstra A Potent hepatitis B core-specific B cell responses associate with clinical parameters in untreated and virally suppressed chronic HBV patients. J Hepatol. (2019) 70:E455 10.1016/S0618-8278(19)30898-9 32061650

[17] Hees SV Hou J Grooshuismink A Kreefft K Bourgeois S Boonstra A Transcriptomic profiling of intrahepatic B cells suggests a B-cell impairment in the immune active phase of chronic hepatitis B. J Hepatol. (2018) 68:S792 10.1016/S0168-8278(18)31855-5

[18] van de Garde MDB Pas SD van Oord GW Gama L Choi Y de Man RA. Interferon-alpha treatment rapidly clears Hepatitis E virus infection in humanized mice. Sci Rep. (2017) 7:8267. 10.1038/s41598-017-07434-y 28811492 PMC5557905

[19] Sari G Yin X Boonstra A Feng ZD Vanwolleghem T Dissecting the different roles of ORF3 in HEV spread and fecal shedding in a humanized mouse model. J Hepatol. (2019) 70:E97–E97. 10.1016/S0618-8278(19)30172-0

